# Elution of gentamicin and vancomycin from polymethylmethacrylate beads and hip spacers in vivo

**DOI:** 10.3109/17453670902884700

**Published:** 2009-04-01

**Authors:** Konstantinos Anagnostakos, Philippe Wilmes, Eduard Schmitt, Jens Kelm

**Affiliations:** Klinik für Orthopädie und Orthopädische Chirurgie, Universität des SaarlandesHomburg/SaarGermany

## Abstract

**Background and purpose** Late infections after total hip arthroplasty are still a problem. Treatment procedures include resection arthroplasty with implantation of antibiotic-loaded beads or implantation of an antibiotic-impreganted spacer. However, little is known about antibiotic elution from bone cement beyond the first 2–3 postoperative days in humans.

**Methods** 17 hip spacers (80g PMMA, 1g gentamicin, and 4 g vancomycin) and 11 chains (40 g PMMA, 0.5 g gentamicin, and 2 g vancomycin) in 28 patients were studied. The release of both agents was measured in the drainage fluid on a daily basis. The drains were left in situ until less than 50 mL was produced per day. The elution of both antibiotics was determined by fluorescence polarization immunoassay. Systemic antibiotics were given postoperatively according to antibiogram. If possible, no gentamicin or vancomycin was given.

**Results** Peak mean concentrations from beads and spacers were reached for gentamicin (1,160 (12–371) µg/mL and 21 (0.7–39) µg/mL, respectively) and for vancomycin (80 (21–198) µg/mL and 37 (3.3–72) µg/mL) on day 1. The last concentrations to be determined were 3.7 µg/mL gentamicin and 23 µg/mL vancomycin in the beads group after 13 days, and 1.9 µg/mL gentamicin and 6.6 µg/mL vancomycin in the spacer group after 7 days. Between the fifth and seventh day, an intermittent increase in elution of vancomycin from both beads and spacers and of gentamicin from spacers was noticed. No renal or hepatic dysfunction was observed.

**Interpretation** Beads showed higher elution characteristics in vivo than the spacers due to their larger surface area; however, a great amount of inter-subject variability was seen for both beads and spacers. The inferior elution properties of spacers emphasize the importance of additional systemic antibiotics for this treatment procedure during the postoperative period. Future studies should clarify whether the dose of antibiotics or length of antibiotic therapy may be reduced in the case of bead implantation, without jeopardizing the control of infection.

## Introduction

Late infections after total hip arthroplasty (THA) still remain a problem. Usually, a two-stage procedure is chosen for treatment of infection, whereby either a resection arthroplasty (Girdlestone procedure) is performed or an antibiotic-loaded spacer is inserted (Hsieh et al. 2004, Anagnostakos et al. 2006).

Recent reports have emphasized the importance of studying and understanding the elution of antibiotics from beads and spacers in such treatment procedures, in order to define the ideal moment for explantation of beads or spacer and to plan for reimplantation of the prosthesis (Bertazzonni Minelli et al. 2004, Anagnostakos et al. 2006). The main advantage of these devices is that antibiotics are released from polymethylmethacrylate (PMMA) in such a way that the local levels of antibiotic vastly exceed the minimum inhibitory or bactericidal concentrations needed to treat most pathogenic organisms, and that these levels are much higher than those achieved with parenteral therapy (Masri et al. 1998). Such high concentrations are important, especially when gentamicin is used for impregnation of bone cement, because this aminoglycoside has a peak dose effect for bactericidal activity (McLean et al. 1993). Isiklar et al. (1999) reported that 57 µg/mL vancomycin could be measured in the drainage fluid from a vancomycin-loaded hip spacer on the first postoperative day; however, later antibiotic release was not measured. Chohfi et al. (1998) used vancomycin-loaded cement for prosthesis fixation in primary THA and determined the concentrations released into the drainage fluid. Bactericidal concentrations could be measured over the first 4 days, but further concentrations were not known because all patients had had their drain removed at this time. In dogs, Adams et al. (1992) found detectable concentrations of tobramycin and vancomycin from beads for 28 days. In rabbits, antibiotic-loaded cement pellets were found to elute antibiotics for up to 37 days (Chapman and Hadley 1976). Jenny et al. (1995) measured gentamicin concentrations in the drainage fluids of patients, but these were measured only on the first postoperative day.

We conducted a prospective study to evaluate the local antibiotic concentrations of gentamicin and vancomycin in patients undergoing two-stage exchange for infected THA: either by inserting antibiotic-loaded beads in the case of a Girdlestone procedure, or by implantation of an antibiotic-loaded spacer.

## Patients and methods

We studied 17 hip spacers (80 g PMMA, 1 g gentamicin, and 4 g vancomycin) (Refobacin/Palacos: Merck, Darmstadt, Germany; Vanco-cell: Cell-Pharm, Hannover, Germany) and 11 chains (40 g PMMA, 0.5 g gentamicin, and 2 g vancomycin) (Refobacin/Palacos, Vanco-cell) in 28 patients (spacer group: 10 males and 7 females, mean age 69 (60–81) years; beads group: 6 males and 5 females, mean age 67 (41–76) years). In all cases, a causative pathogenic organism could be identified in the wound. In the spacer group, *S. epidermidis* was isolated 6 times, *S. aureus* 5 times, a methicillin-resistant *S. aureus* (MRSA) 3 times, and there were single isolates of *P. mirabilis, E. faecalis*, and *E. faecium.* In the beads group, *S. epidermidis, S. aureus* and MRSA were isolated 3 times each and there were single isolates of *S. haemolyticus* and *E. faecalis.* All organisms were sensitive to both gentamicin and vancomycin, except for the MRSA strains, which were gentamicin-resistant. Both spacers (head diameter: 50 mm; stem length: 10 cm; surface area: 133 cm^2^) and chains (40 beads, total length: 93 cm; surface area: 160 cm^2^) were produced intraoperatively using molds, as reported previously (Kelm et al. 2004, Anagnostakos et al. 2005). In short, vancomycin was thoroughly mixed by hand into Refobacin/Palacos powder. After about 5 min of mixing, the cement's liquid monomer was added. The cement dough was then poured into the two halves of the mold for the spacer/beads. The halves were then clamped together and, after about 15 min, they were opened and the molded spacer/chain was removed.

In all cases, the primary indication for implantation of spacers and beads was late infection after total hip arthroplasty. The choice of bead or spacer implantation was made according to the general condition of each patient. If a patient was compromised by his general medical condition and thus could not afford a later prosthesis reimplantation, or was not willing to undergo a two-stage procedure, a Girdlestone arthroplasty with insertion of beads was performed. In each of these cases, half of the chain produced was inserted into the femur and the other half was inserted into the acetabular cavity. In the other cases, an articulated antibiotic-loaded spacer was implanted. All spacers were fixed to the femur by cementation to its proximal part. In all cases, the bone cement was loaded with gentamicin and vancomycin due to the broad antimicrobial spectrum and the known synergistic effect of these 2 agents regarding elution (Anagnostakos et al. 2006).

**Figure 1.**
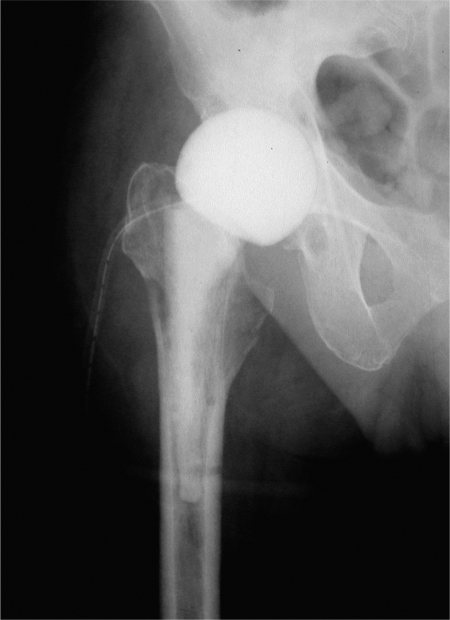
Gentamicin- and vancomycin-impregnated spacer in situ. The elution of antibiotics was determined using the wound drainage placed at the head of the spacer.

Over the first postoperative days, a wound secretion specimen was taken from the redon drainage bottle every 24 h via the drainages placed directly at the head of the spacers or near the beads (Figure 1). The redons were left in situ until less than 50 mL/day was produced. For the spacers, redons were left in situ for a maximum of 7 days, and for 13 days in the case of beads. The drainage system was changed daily. A systemic antibiotic was given in each case according to antibiogram, but no patients were given gentamicin or vancomycin intravenously in order to ensure that the concentrations measured in the drainage fluid could only be attributed to elution from the cement.

A fluorescence polarization immunoassay (FPIA) (Abbot, Wiesbaden, Germany) was used to determine the concentrations of gentamicin and vancomycin released. The lowest measurable concentration of drug was 0.27 µg/mL for gentamicin and 2.0 µg/mL for vancomycin.

## Results

**Figure 2.**
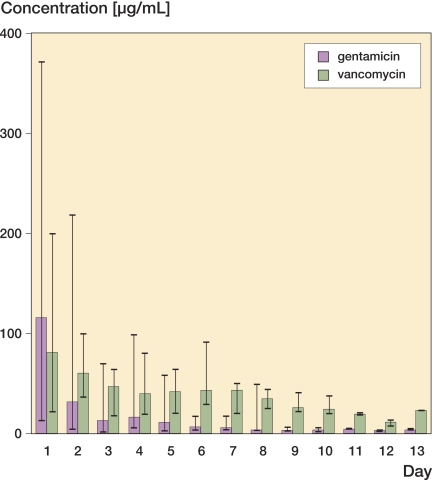
Elution of gentamicin and vancomycin from beads over the first 13 postoperative days. Mean and extreme values (minimum-maximum) are shown for each day.

**Figure 3.**
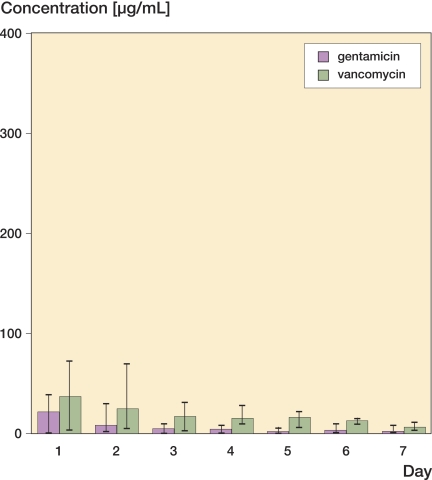
Elution of gentamicin and vancomycin from spacers over the first 7 postoperative days. Mean and extreme values (minimum-maximum) are shown for each day.

Both agents showed similar release characteristics over the first postoperative days (Figures 2 and 3). In the beads group, mean gentamicin concentration was higher than that of vancomycin on day 1 (116 (12–371) µg/mL vs. 80 (21–198) µg/mL, respectively), whereas in the days that followed the mean concentration of vancomycin was always higher than that of gentamicin. In the spacer group, mean vancomycin concentrations were higher than those of gentamicin on day 1 (37 (3.3–72) µg/mL vs. 21.1 (0.7–39) µg/mL) and remained higher over the entire length of the measurement period.

**Figure 4.**
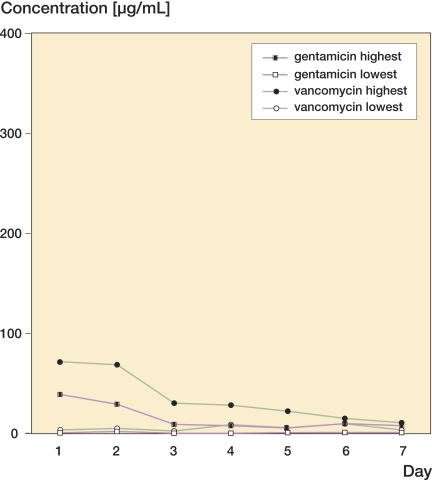
The elution from the spacers showed substantial inter-individual variability in the peak amounts of antibiotic released during the first postoperative days.

**Figure 5.**
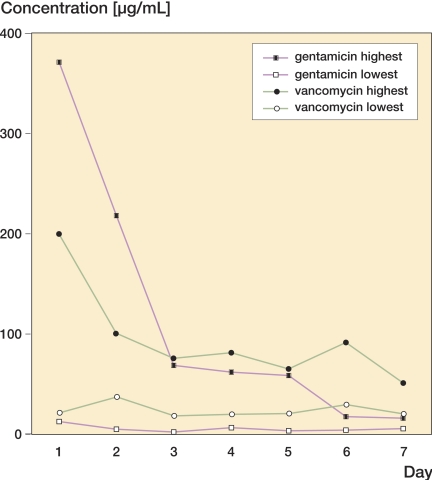
The release of antibiotic from beads also showed substantial inter-individual variability for both agents regarding the peak amounts of antibiotic released during the first postoperative days.

After reaching their highest concentrations on day 1, the elution of both antibiotics decayed constantly at a similar rate over the following days. . The last concentrations to be determined were 3.7 µg/mL gentamicin and 23 µg/mL vancomycin in the beads group after 13 days, and 1.9 µg/mL gentamicin and 6.6 µg/mL vancomycin in the spacer group after 7 days. Between the fifth and the seventh day, we found an intermittent increase in the elution of vancomycin from both beads and spacers and of gentamicin from spacers. There was a substantial degree of inter-subject variability with regard to the peak amounts released (Figures 4 and 5).

No renal or hepatic dysfunction was observed in any of the cases.

The total amount of gentamicin and vancomycin released from beads and spacers was not determined.

## Discussion

Since the introduction of antibiotic-loaded bone cement by Buchholz and Engelbrecht in the early 1970s (Buchholz and Engelbrecht 1970), antibiotic-impregnated bone cement has been used in the prophylaxis and treatment of orthopedic infections by means of spacers or beads (Klemm 1976, Anagnostakos et al. 2006).

Due to the increasing emergence of multiresistant pathogenic organisms over the past few years, cement media loaded with two different antibiotics are being used more frequently in the treatment of orthopedic infections (Masri et al. 1998, Anagnostakos et al. 2006). It is known that there is a synergistic effect between aminoglycosides and glycopeptides with regard to antimicrobial properties and pharmacokinetics of bone cement (Anagnostakos et al. 2005), but few data are available on the precise nature of the interactions between these substances in vivo. Klekamp et al. (1999) studied the release of tobramycin and vancomycin from Simplex and Palacos cement in vitro and found that the release of tobramycin was compromised by the presence of vancomycin, whereas the release of vancomycin was not compromised by the presence of tobramycin. Another in vitro study by Penner et al. (1996) demonstrated that the combination of both antibiotics in PMMA enhances the release of tobramycin by 68% and of vancomycin by 103% in comparison to controls containing tobramycin or vancomycin alone. In vivo studies of tobramycin- and vancomycin-impregnated hip and knee spacers showed superior elution for tobramycin than for vancomycin, but the elution of vancomycin was enhanced by the presence of tobramycin (Masri et al. 1998).

We have not found any previous studies describing measurement of antibiotic elution from PMMA on a daily basis beyond the first 2–3 postoperative days. The greater amount of vancomycin incorporated into PMMA should explain our findings of higher concentrations released over the early postoperative period, except for the first day. Despite the fact that the beads were impregnated with half of the amount of antibiotic that the spacers were, in the first days the beads released greater amounts of both agents due to their larger surface area (Anagnostakos et al. 2006). Although the difference between the surface area of the chains and that of the spacer is not appreciable, we would explain this discrepancy by the fact that most of the antibiotic-releasing surface of each spacer is covered by the proximal part of the femur where the antibiotics cannot be washed off from the surface as easily as in the case of beads.

Hsieh et al. (2006) reported recently on the elution of vancomycin and aztreonam from hip spacers. Peak concentrations of vancomycin initially were 1,538 µg/mL and they decreased over 7 days to a mean value of 519 µg/mL. However, the spacer was loaded with 4 g vancomycin per 40 g PMMA whereas ours contained only 2 g vancomycin per 40 g PMMA. Furthermore, aztreonam may have a different influence on the pharmacokinetics of vancomycin than gentamicin has; thus, another synergistic effect might result.

Nelson et al. (1994) studied the residual elution properties of gentamicin-loaded beads in vitro after retrieval. They found that beads that had been implanted for less than 10 weeks released significantly higher levels of antibiotics than beads that had been implanted for more than 14 weeks. Interestingly, there also exist in vitro findings on explanted spacers that indicate different residual capacities with regard to antibiotic release and antimicrobial properties independently of the implantation period (Bertazzonni Minelli et. al. 2004). Although the exact cause of this discrepancy between published data is unclear, it is possible that individual variations in the local perfusion during implantation, anatomical variations and changes in the cement surface due to coverage, e.g. from scar tissue, may play a role. The tissue penetration and absorption of both agents in vivo might also be an issue.

Our results also show high inter-subject variability with regard to the peak concentrations of antibiotic measured. For example, in the beads group the mean gentamicin concentration was 116 µg/mL on day 1, while the maximum concentration measured was at 370 µg/mL. Moreover, peaks were measured between the fifth and seventh day. This discrepancy may be attributable to several factors such as local blood perfusion or pH value of the tissues; however, we believe that the manual incorporation of the vancomycin into the cement powder is the main parameter responsible for the variability. This inhomogenity of antibiotic elution has been seen by the authors in in vitro studies (Anagnostakos et al. 2005) and can often be expected with the use of non-commercially manufactured antibiotic-loaded cement media. Furthermore, this inhomogeneity combined with the above-listed inter-individual factors makes it difficult to assess with any accuracy the total amount of antibiotic released from spacers in vivo. Bertazzonni Minelli et al. (2004) have shown that the release of antibiotic is still irregular even 3–4 months after hip spacer implantation, independently of the implantation time.

The fact that a sufficient amount of antibiotic release from beads lasts longer than from spacers emphasizes the importance of systemic antibiotics, given as a complement to the spacer implantation, over the first 4–6 postoperative weeks in order to avoid not only spread of the infection, but also persistence of the infection locally. Perhaps in the case of a bead implantation, the dose of the intravenous antibiotic could be reduced over the first 2 postoperative weeks due to sufficiency of local antibiotic therapy, but after this time period a re-adjustment of the dose might be necessary in order to prevent the emergence of resistant bacterial strains—since the local antibiotic concentrations decrease over time. Future clinical studies should clarify this issue. Some antibiotics require measurement of peak and trough serum levels during therapy, and the dose is usually adjusted based on those levels. Generally, in the case of late infections where the source of the infection is blood-based, the use of systemic antibiotics is important—not only for treatment of the joint infection, but also for treatment of the primary source of the infection and to avoid any other manifestations of infection. However, in the presence of antibiotic-loaded cement devices, complications might occur despite the fact that such media are considered to be safe (Salvati et al. 1986). Side effects are probably the result of systemic antibiotic administration along with local therapy. Koo et al. (2001) described the occurrence of a transient liver dysfunction and bone marrow depression following the simultaneous implantation of an antibiotic-loaded spacer and intravenous treatment with antibiotics. Patrick et al. (2006) described an acute renal failure after the use of vancomycin- and tobramycin-loaded spacers in total hip arthroplasty.

In summary, gentamicin- and vancomycin-loaded beads and hip spacers appear to be capable of releasing sufficient concentrations over the first 7–13 postoperative days in vivo. Future studies will be required to determine whether this also applies to other antibiotics and other amounts of antibiotic incorporated into PMMA. We recommend the use of systemic antibiotics for at least the first 4 postoperative weeks, to ensure eradication of the infection and to avoid re-infections or persistent infections, especially since both beads and spacers have shown an appreciable amount of inter-individual variability regarding peak concentrations of antibiotic release.

## References

[CIT0001] Adams K, Couch L, Cierny G, Calhoun J, Mader JT (1992). In vitro an din vivo evaluation of antibiotic diffusion from antibiotic-impregnated polymethylmethacrylate beads.. Clin Orthop.

[CIT0002] Anagnostakos K, Kelm J, Regitz T, Schmitt E, Jung W (2005). In vitro evaluation of antibiotic release from and bacterai growth inhibition by antibiotic-loaded acrylic bone cement spacers.. J Biomed Mater Res B Appl Biomater.

[CIT0003] Anagnostakos K, Fürst O, Kelm J (2006). Antibiotic-impregnated hip spacers: Current status.. Acta Orthop.

[CIT0004] Bertazzonni Minelli E, Benini A, Magnan B, Bartolozzi P (2004). Release of gentamicin and vancomycin from temporary human hip spacers in two-stage revision of infected arthroplasty.. J Antimicrob Chemother.

[CIT0005] Buchholz HW, Engelbrecht H (1970). Depot effects of various antibiotics mixed with Palacos resins.. Chirurg.

[CIT0006] Chapman MW, Hadley WK (1976). The effect of polymethylmethacrylate and antibiotic combinations on bacterial viability. An in vitro and preliminary in vivo study.. J Bone Joint Surg (Am).

[CIT0007] Chohfi M, Langlais F, Fourastier J, Minet J, Thomazeau, Cormier M (1998). Pharmacokinetics, uses, and limitations of vancomycin-loaded bone cement.. Int Orthop.

[CIT0008] Hsieh PH, Shih CH, Chang YH, Lee MS, Shih HN, Yang WE (2004). Two-stage revision hip arthroplasty for infection: comparison between the interim use of antibiotic-loaded cement beads and a spacer prosthesis.. J Bone Joint Surg (Am).

[CIT0009] Hsieh PH, Chang YH, Chen SH, Ueng SW, Shih CH (2006). High concentration and bioactivity of vancomycin and aztreonam eluted from Simplex cement spacers in two-stage revision of infected hip implants: a study of 46 patients at an average follow-up of 107 days.. J Orthop Res.

[CIT0010] Isiklar ZU, Demirors H, Akpinar S, Tandogan RN, Alparslan M (1999). Two-stage treatment of chronic staphylococcal orthopaedic implant-related infections using vancomycin impregnated PMMA spacer and rifampicin containing antibiotic protocol.. Bull Hosp Jt Dis.

[CIT0011] Jenny JY, Jenny G, Lambert J, Gaudias J, Kempf I (1995). Utility of measurement of gentamicin release from PMMA beads in wound drainage fluid after in-vivo implantation.. Acta Orthop Belg.

[CIT0012] Kelm J, Anagnostakos K, Regitz T, Schmitt E, Schneider G, Ahlhelm F (2004). MRSA-infections-treatment with intraoperatively produced gentamicin-vancomycin PMMA beads.. Chirurg.

[CIT0013] Klekamp J, Dawson JM, Haas DW, Deboer D, Christie M (1999). The use of vancomycin and tobramycin in acrylic bone cement: biomechanical effects and elution kinetics for use in joint arthroplasty.. J Arthroplasty.

[CIT0014] Klemm K (1976). Treatment of chronic bone infection with gentamicin PMMA-chains and beads.. Accid Surg.

[CIT0015] Koo K-H, Yang J-W, Cho S-H, Song H-R, Park H-B, Ha Y-C, Chang J-D, Kim S-Y, Kim Y-H (2001). Impregnation of vancomycin, gentamicin and cefotaxime in a cement spacer for two-stage cementless reconstruction in infected total hip arthroplasty.. J Arthroplasty.

[CIT0016] Masri BA, Duncan CP, Beauchamp CP (1998). Long-term elution of antibiotics from bone-cement. An in-vivo study using the prosthesis of antibiotic-loaded acrylic-cement (PROSTALAC).. J Arthroplasty.

[CIT0017] McLean AJ, Ioannidesdemos LL, Li SC, Bastone EB, Spicer WJ (1993). Bactericidal effect of gentamicin peak concentration provides a rationale for administration of bolus doses.. J Antimicrob Chemother.

[CIT0018] Nelson CL, Hickmon SG, Harrison BH (1994). Elution characteristics of gentamicin-PMMA beads after implantation in humans.. Orthopedics.

[CIT0019] Patrick BN, Rivey MP, Allington DR (2006). Acute renal failure associated with vancomycin- and tobramycin-laden cement in total hip arthroplasty.. Ann Pharmacother.

[CIT0020] Penner MJ, Masri BA, Duncan CP (1996). Elution characteristics of tobramycin and vancomycin combined in acrylic bone cement.. J Arthroplasty.

[CIT0021] Salvati EA, Callaghan JJ, Brause BD, Klein RF, Small RD (1986). Reimplantation in infection. Elution of gentamicin from cement and beads.. Clin Orthop.

